# Comparing bowel and urinary domains of patient‐reported quality of life at the end of and 3 months post radiotherapy between intensity‐modulated radiotherapy and proton beam therapy for clinically localized prostate cancer

**DOI:** 10.1002/cam4.3414

**Published:** 2020-09-15

**Authors:** Miao Bai, Kimberly R. Gergelis, Mustafa Sir, Thomas J. Whitaker, David M. Routman, Bradley J. Stish, Brian J. Davis, Thomas M. Pisansky, Richard Choo

**Affiliations:** ^1^ Department of Operations and Information Management University of Connecticut Storrs CT USA; ^2^ Department of Radiation Oncology Mayo Clinic Rochester MN USA; ^3^ Department of Health Sciences Research Mayo Clinic Rochester MN USA; ^4^ Department of Radiation Oncology Baylor College of Medicine Houston TX USA

**Keywords:** EPIC‐26, intensity modulated radiotherapy, prostate cancer, proton beam radiotherapy, quality of life

## Abstract

**Purpose:**

To prospectively assess acute differences in patient‐reported outcomes in bowel and urinary domains between intensity‐modulated radiotherapy (IMRT) and proton beam therapy (PBT) for prostate cancer.

**Methods and Materials:**

Bowel function (BF), urinary irritative/obstructive symptoms (UO), and urinary incontinence (UI) domains of EPIC‐26 were collected in patients with T1‐T2 prostate cancer receiving IMRT or PBT at a tertiary cancer center (2015‐2018). Mean changes in domain scores were analyzed from pretreatment to the end of and 3 months post‐radiotherapy for each modality. A clinically meaningful change was defined as a score change >50% of the baseline standard deviation.

**Results:**

A total of 157 patients receiving IMRT and 105 receiving PBT were included. There were no baseline differences in domain scores between cohorts. At the end of radiotherapy, there was significant and clinically meaningful worsening of BF and UO scores for patients receiving either modality. In the BF domain, the IMRT cohort experienced greater decrement (−13.0 vs −6.7, *P* < .01), and had a higher proportion of patients with clinically meaningful reduction (58.4% vs 39.5%, *P* = .01), compared to PBT. At 3 months post‐radiotherapy, the IMRT group had significant and clinically meaningful worsening of BF (−9.3, *P* < .001), whereas the change in BF score of the PBT cohort was no longer significant or clinically meaningful (−1.2, *P* = .25). There were no significant or clinically meaningful changes in UO or UI 3 months post‐radiotherapy.

**Conclusions:**

PBT had less acute decrement in BF than IMRT following radiotherapy. There was no difference between the two modalities in UO and UI.

## Graphical abstract

Prospectively‐collected EPIC‐26 scores were analyzed to assess differences in patient‐reported outcomes in bowel and urinary domains between patients treated with intensity‐modulated radiotherapy (IMRT) and proton beam therapy (PBT) for localized prostate cancer at the end of and 3 months post‐radiotherapy at a tertiary cancer center. Patients treated with PBT had less acute decrement in the bowel function domain than those treated with IMRT; there was no difference between the two modalities in the urinary irritative/obstructive and urinary incontinence (UI) domains.

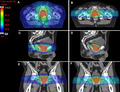

## 1 INTRODUCTION

As patients treated for prostate cancer often survive many years, the relevant outcomes of their treatment include not only the oncologic result, but also the treatment‐related toxicity and overall quality of life (QOL). Studies have documented that both radical prostatectomy and radiotherapy (RT) can result in temporary and permanent changes to bladder, bowel, and sexual function.^1^ Thus, minimizing treatment‐related toxicity and preserving QOL have been key components of patients’ decision‐making when choosing a treatment modality for prostate cancer.

Technologic advances have been made over the years to conform RT dose more closely to a target. The availability of more conformal RT has enabled clinicians to increase dose to the prostate and, at the same time, reduce overall dose to adjacent normal organs such as the rectum and bladder. This differential dose distribution can maximize tumor control, while minimizing potential adverse effects of RT. Such conformal RT modalities include intensity modulated RT (IMRT) and proton beam therapy (PBT).

PBT has been increasingly used as a means to potentially further improve the therapeutic ratio of external beam RT (EBRT) for the treatment of clinically localized prostate cancer. Unlike photons, protons have a distinctive physical property that allows the deposition of most of their energy to the depth of a specified target with very little exit dose beyond the target. This unique dose‐deposition characteristic helps protons more readily achieve a delicate balance of delivering a high radiation dose to the prostate with less dose to adjacent normal organs, compared with photon‐based IMRT.

There has been no published phase III study comparing PBT to IMRT for the treatment of prostate cancer with respect to tumor control or radiation morbidity, although several single‐institution studies of PBT reported promising results.^2^, ^3^ At present, two phase III studies comparing PBT with IMRT are in progress (ClinicalTrials.gov Identifiers: NCT01617161, NCT04083937). In the Prostate Advanced Radiation Technologies Investigating Quality of Life (PARTIQoL) and Prostate Cancer Patients Treated with Alternative Radiation Oncology Strategies (PAROS) trials, the primary endpoint is to assess whether PBT is superior to IMRT in patient‐reported bowel function. More comparative studies are needed to address whether PBT provides a clinically meaningful benefit.

At our institution, patients opting for EBRT for the treatment of clinically localized prostate cancer may receive either IMRT or PBT as standard of care. All patients receiving definitive EBRT with either IMRT or PBT have been encouraged to participate in a prospective registry to evaluate radiation‐related toxicity and the impact of RT on QOL.

The purpose of this research is to evaluate whether patient‐reported QOL differs in bowel and urinary domains between IMRT and PBT at the end of RT and 3 months post‐RT, using prospectively collected 26‐item Expanded Prostate Index Composite (EPIC‐26) data.

## 2 METHODS

### 2.1 Selection of participants

This study's cohort was comprised of patients identified using an IRB‐approved institutional prospective registry who received IMRT or PBT to the prostate ±the proximal seminal vesicles for clinical stage T1‐T2 N0 M0 prostate cancer at a single tertiary cancer center between April 2015 and March 2018 and completed the EPIC‐26 questionnaire prior to the start of RT. Patients receiving elective pelvic nodal RT were excluded. The study focused on patients treated with one of the three dose‐fractionation regimens: 60 Gy in 20 fractions, 70.2 Gy in 26 fractions, or 78 Gy in 39 fractions. These dose‐fractionation regimens were more widely used than any other regimen during this period. Other dose‐fractionation regimens were excluded to reduce confounding. All patients were participants of the IRB‐approved registry for prospective evaluation of patient‐reported toxicity and QOL.

### 2.2 Measurement of patient‐reported quality of life

Patient‐reported QOL was assessed by the EPIC‐26 questionnaire. This instrument includes domain scores for bowel function (BF), urinary irritative/obstructive symptoms (UO), urinary incontinence (UI), sexual function (SF), and hormonal function (HF). Patients were asked to complete the EPIC‐26 questionnaire prior to RT (baseline), at the end of RT, and at 3, 6, 12, 24, 36, and 48 months after the completion of RT. Responses of each domain were scored from 0‐100, as conventionally processed, with higher scores indicating better function.^4^

The primary outcomes examined in this study were the change in the scores of BF, UO, and UI domains from pretreatment to the end of RT and 3 months post‐RT, assessing the early effects of RT on these domains. SF and HF domains were not included in the analysis as they were confounded by the use of androgen deprivation therapy (ADT).

### 2.3 Treatment details

**FIGURE 1:**
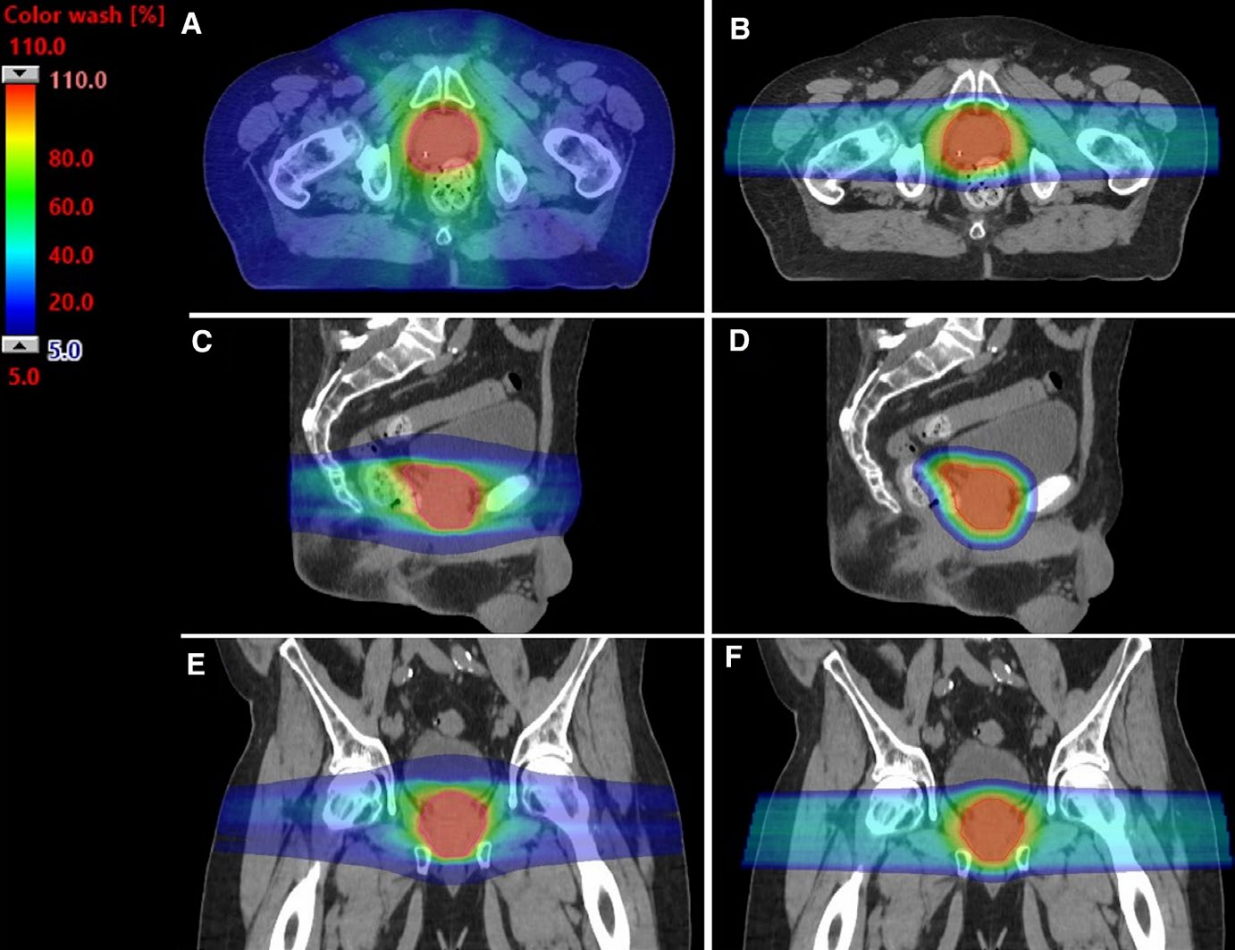
Representative IMRT and PBT plans. Intensity modulated radiotherapy (IMRT) and pencil beam scanning proton beam radiotherapy (PBT) plans for a patient with localized prostate cancer treated with 7800 cGy in 39 fractions. Panels A&B show axial slices of IMRT (A) and PBT (B); panels C&D show sagittal slices of IMRT (C) and PBT (D); panels E&F show coronal slices of IMRT (E) and PBT (F). Red and orange indicate higher doses (70‐80 Gy), green indicates medium doses (40‐60 Gy), and blue indicates lower doses (10‐30 Gy). Note sparing of rectum and bladder with PBT vs IMRT

At our institution, PBT is delivered by intensity‐modulated pencil beam scanning with spot spacing of 3 mm, typically using two lateral beams or two lateral plus two anterior oblique beams. IMRT is delivered utilizing volumetric modulated arc therapy, typically with two arcs. A clinical target volume to planning target volume margin of 4‐5 mm is utilized with both PBT and IMRT plans (Figure 1).

### 2.4 Statistical analysis

We initially examined any differences in the baseline characteristics, including demographic and clinical features, and baseline EPIC‐26 scores in BF, UO, and UI domains between patients treated with IMRT and those receiving PBT. This was done to assess whether a meaningful analysis could be performed regarding the potential differences in the extent of QOL change associated with the two treatment modalities. In addition, we compared the baseline characteristics between the responders and the non‐responders to the 3‐month EPIC‐26 questionnaire to assess whether the outcomes obtained from the responders can be generalized to the overall patient population undergoing definitive EBRT at our institution.

For the evaluation of the early effects of RT on patient‐reported QOL, we examined the mean changes in EPIC‐26 scores from the baseline to the end of treatment and 3 months post‐RT for each RT modality separately. The evaluation of the changes in EPIC‐26 scores was limited to patients who completed the questionnaire both at baseline and at the specified time point of interest.

To assess the difference in the score changes in BF, UO, and UI domains between IMRT and PBT, the extent of the score changes in these domains from baseline to a specified time point (at the end of RT and 3 months post‐RT) was compared between the patients treated with IMRT and those receiving PBT. In addition, for each RT modality, we assessed the statistical significance and clinical relevance of score changes at the end of RT and 3 months post‐RT. A clinically meaningful change at a specified point of time was defined as a score change that exceeds >50% of the standard deviation of a baseline score.^5^, ^6^, ^7^ The difference in the proportion of patients with clinically meaningful change between the two treatment modalities was also evaluated.

To identify variables that significantly impacted EPIC‐26 score changes over time, we evaluated the independent effects of multiple variables. Variables examined were the RT modality (IMRT vs PBT), dose‐fractionation regimen utilized (60 Gy in 20 vs 70.2 Gy in 26 vs 78 Gy in 39), use of a rectal hydrogel spacer (yes vs no), baseline BF, UO, and UI scores, age, race, pre‐RT prostate‐specific antigen (PSA), Gleason score, T stage, and the use of ADT. We used the generalized estimating equation (GEE), a semiparametric extension of generalized linear model, to handle unknown correlation between EPIC‐26 scores at the end of RT and those at 3 months post‐RT.^8^

Two‐sided Wilcoxon rank‐sum test, Wilcoxon signed‐rank sum test, Fisher's exact test, and Wald test were used for testing of statistical hypotheses with *P* < .05 considered statistically significant unless specified otherwise. For analyses involving multiple pairwise comparisons, *P* < .017 was considered statistically significant to account for Bonferroni adjustment.

## 3 RESULTS

### 3.1 Baseline characteristics and EPIC – 26 questionnaire completion rate

A total of 157 patients treated with IMRT and 105 patients treated with PBT met the study inclusion criteria and were the basis of this study. Patient characteristics are depicted in Table 1. There were no differences between the IMRT and PBT cohorts with respect to mean age, race, T stage, Gleason score, mean PSA, PSA distribution, and the proportion of patients treated with hydrogel spacer. Patients treated with IMRT were more likely to receive ADT than those receiving PBT (80.9% vs 67.6%, *P* = .02). The proportion of patients receiving the three different dose‐fractionation regimens in the IMRT cohort vs the PBT cohort were 47.8% vs 31.4% for 60 Gy in 20 fractions, 42.7% vs 52.4% for 70.2 Gy in 26 fractions, and 9.6% vs 16.2% for 78 Gy in 39 fractions.

**TABLE 1:** Baseline characteristics of IMRT and PBT cohorts

Characteristics	IMRT (n = 157)	PBT (n = 105)	*P*‐value*
Age			
Mean (range), year	71.5 (54‐84)	70.4 (44‐88)	.19
<70	58 (36.9%)	43 (41.0%)	.78
70‐79	90 (57.3%)	56 (53.3%)	
≥80	9 (5.7%)	6 (5.7%)	
Race			
White	148 (94.3%)	100 (95.2%)	.99
Others	7 (4.5%)	4 (3.8%)	
Missing	2 (1.3%)	1 (1.0%)	
Pre‐RT PSA			
Mean (range, ng/mL)	7.3 (0.1‐39.4)	7.9 (0.1‐41.7)	.29
<4	48 (30.6%)	22 (21.0%)	.22
4‐10	73 (46.5%)	55 (52.4%)	
>10	36 (22.9%)	28 (26.7%)	
Gleason score			
≤7	135 (86.0%)	89 (84.8%)	.86
>7	22 (14.0%)	16 (15.2%)	
T stage			
T1	76 (48.4%)	41 (39.0%)	.16
T2	81 (51.6%)	64 (61.0%)	
Dose‐fractionation			
60 Gy in 20	75 (47.8%)	33 (31.4%)	**.02**
70.2 Gy in 26	67 (42.7%)	55 (52.4%)	
78 Gy in 39	15 (9.6%)	17 (16.2%)	
Hydrogel spacer			
Hydrogel spacer use, n (%)	77 (49.0%)	47 (44.8%)	.53
Androgen deprivation therapy (ADT)			
Treated with ADT, n (%)	127 (80.9%)	71 (67.6%)	**.02**
Baseline bowel function score			
Mean (range)	93.9 (29.2‐100)	93.9 (50‐100)	.64
Standard deviation	9.9	10.6	
Missing	5	1	
Baseline urinary irritative/obstructive score			
Mean (range)	85.7 (31.3‐100)	85.4 (43.8‐100)	.68
Standard deviation	13.8	12.9	
Missing	5	6	
Baseline urinary incontinence score			
Mean (range)	87.6 (22.8‐99.5)	87.7 (39.3‐99.5)	.89
Standard deviation	16.6	16.1	
Missing	0	2	

Bolded values are statistically significant.

* *P*‐values were derived from Wilcoxon rank‐sum test for continuous variables, and Fisher's exact test for categorical variables. *P*‐values reflect a test whether the means or the distributions were different between the two cohorts. *P* < .05 is considered significant.

The response rates of completing the EPIC‐26 questionnaire at the end of RT and 3 months post‐RT were 86.3% and 49.2%, respectively. There was no statistical difference in the completion rates between the IMRT and PBT cohorts. In addition, no statistically significant difference was found with respect to demographics, clinical characteristics, and baseline EPIC‐26 scores between the patients who completed the questionnaire and those that did not (Table A1 in appendix).

### 3.2 Comparison of the changes in the scores of BF, UO and UI domains between IMRT and PBT cohorts

At baseline, there were no statistically significant differences in the scores of BF, UO, and UI domains between the IMRT and PBT cohorts, as shown in Table 1.

**FIGURE 2:**
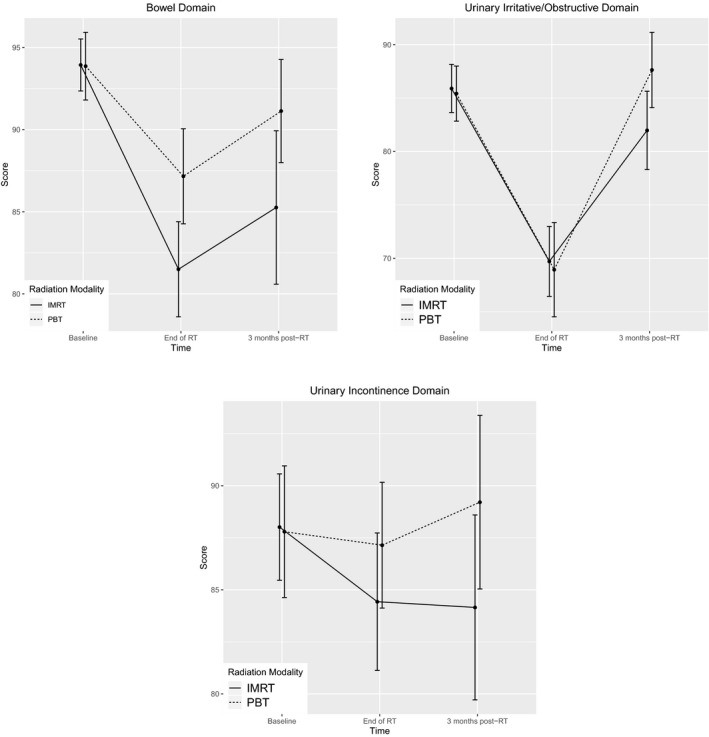
Score changes in mean bowel function, urinary irritative/obstructive and urinary incontinence domains from baseline to the end of RT and 3 months post‐RT with 95% confidence interval

Figure 2 and Table 2 describe the changes in the BF, UO, and UI domains at the end of RT and 3 months post‐RT in the IMRT cohort vs the PBT cohort. In both cohorts, the scores of BF, UO, and UI domains declined at the end of treatment, but improved at 3 months post‐RT (Figure 2). Compared to the PBT cohort, the IMRT cohort experienced greater reduction in BF. This difference was statistically significant at the end of the treatment (*P* < .01) and retained a borderline statistical significance (*P* = .02) at 3 months post‐RT. The differences observed in the UO and UI domains between IMRT and PBT were not statistically significant.

**TABLE 2:** Comparison of score changes at the end of RT and 3 months post‐RT between IMRT cohort and PBT cohort

EPIC‐26 domain	Radiation modality	End of RT		3‐months post RT	
		Mean change from baseline	*P* *	Mean change from baseline	*P* *
Bowel function	IMRT	−13.0	**<.01**	−9.3	**.02**
	PBT	−6.7		−1.2	
Urinary irritative/obstructive	IMRT	−16.2	.98	−2.4	.03
	PBT	−16.4		1.7	
Urinary incontinence	IMRT	−4.3	.23	−2.5	.21
	PBT	−2.6		−0.4	

The value of <.01 represents statistical significance; the value of .02 represents borderline statistical significance (in bold).

* *P*‐values were derived from Wilcoxon rank‐sum test. *P* ^‐^values reflect a test whether score changes were different between two treatment cohorts. *P* < .017 is considered significant to account for Bonferroni correction.

Table 3 describes the score changes in the three domains at the end of RT and 3 months post‐RT for each RT modality and examines whether these score changes are statistically significant and clinically meaningful. At the end of RT, both the IMRT and PBT cohorts showed statistically significant (*P* < .001) and clinically meaningful reduction in BF and UO domains. In the UI domain, the IMRT cohort showed a statistically significant (*P* < .001), but not a clinically meaningful, reduction, while the PBT cohort did not have statistically significant or clinically meaningful reduction. At 3 months post‐RT, a statistically significant (*P* < .001) and clinically meaningful reduction in the BF domain was observed in the IMRT cohort, but not in the PBT cohort. In the UO and UI domains, there was no statistically significant or clinically meaningful reduction in either cohort at 3 months post‐RT.

**TABLE 3:** Score changes and their clinical relevance at the end of RT and 3 months post‐RT for each treatment modality

	EPIC‐26 domain	Radiation modality	No. of respondents (%)	Mean score change from baseline (range)	*P* *	Is mean score change clinically meaningful? (Y/N)**
End of RT	Bowel function	IMRT	125 (79.6%)	−13.0 (−54.2, 20.8)	**<.001**	**Y**
		PBT	86 (81.9%)	−6.7 (−50.0,45.8)	**<.001**	**Y**
	Urinary irritative/obstructive	IMRT	123 (78.3%)	−16.2 (−75.0, 37.5)	**<.001**	**Y**
		PBT	81 (77.1%)	−16.4 (−56.3, 18.8)	**<.001**	**Y**
	Urinary incontinence	IMRT	126 (80.3%)	−4.3 (−47.8, 47.8)	**<.001**	N
		PBT	84 (80.0%)	−2.6 (−39.5, 31.3)	.04	N
3 months post‐RT	Bowel function	IMRT	68 (43.3%)	−9.3 (−91.7, 29.2)	**<.001**	**Y**
		PBT	47 (44.8%)	−1.2 (−25.0, 20.8)	.25	N
	Urinary irritative/obstructive	IMRT	70 (44.6%)	−2.4 (−56.3, 31.3)	.18	N
		PBT	48 (45.7%)	1.7 (−56.3, 25.0)	.12	N
	Urinary incontinence	IMRT	69 (43.9%)	−2.5 (−35.3, 54.0)	.09	N
		PBT	51 (48.6%)	−0.4 (−56.0, 33.3)	.99	N

Bold values are statistically significant or clinically meaningful.

* *P*‐values were derived from Wilcoxon signed‐rank sum test, and reflect a test whether scores at the end of RT and 3 months post‐RT were different from baseline score. *P* < .017 is considered significant to account for Bonferroni adjustment.

** When a score change exceeds >50% of the standard deviation of baseline score, it is considered clinically meaningful.

Table 4 shows whether there was a statistically significant difference in the proportion of patients experiencing clinically meaningful reduction in the three domains between the IMRT and PBT cohorts. In the UO and UI domains, there was no statistically significant difference between the two cohorts. However, in the BF domain, the IMRT cohort had a higher proportion of patients experiencing clinically meaningful reduction compared with the PBT cohort. This difference was statistically significant at the end of RT (*P* = .01) and had borderline significance (*P* = .02) at 3 months post‐RT.

**TABLE 4:** Comparison of the proportions of patients with clinically meaningful changes at the end of RT and 3 months post‐RT between IMRT cohort and PBT cohort

EPIC‐26 domain	Radiation Modality	End of RT		3 months post‐RT	
		% of patients with clinically meaningful reduction	*P* *	% of patients with clinically meaningful reduction	*P* *
Bowel	IMRT	73 (58.4%)	**.01**	27 (39.7%)	**.02**
	PBT	34 (39.5%)		9 (19.1%)	
Urinary irritative/obstructive	IMRT	81 (65.8%)	.37	16 (22.9%)	.23
	PBT	48 (59.3%)		6 (12.5%)	
Urinary incontinence	IMRT	37 (29.4%)	.88	17 (24.6%)	.83
	PBT	23 (27.4%)		11 (21.6%)	

The value .01 is statistically significant; .02 is borderline statistically significant (in bold).

* *P*‐values were derived from Fisher's exact test. *P*‐values reflect a test whether the difference in the proportion of patients experiencing clinically meaningful reduction is statistically significant between two modalities. *P* < .017 is considered significant to account for Bonferroni adjustment.

### 3.3 Variables associated with the changes in the scores of the three domains

Table 5 summarizes the variables significantly associated with the changes in the scores of the three domains over time. Treatment with PBT, in comparison to IMRT, correlated with higher BF score (ie less bowel symptoms) at the end of RT and 3 months post‐RT (*P* < .001). A higher baseline UO score (ie less irritative/obstructive symptoms) (*P* = .01) was correlated with greater reduction in UO domain. A higher baseline UI score (ie less urinary incontinence) (*P* < .001), a lower baseline UO score (ie worse irritative/obstructive symptoms) (*P* = .003), and a higher baseline BF score (ie less bowel symptoms) (*P* = .05) were associated with greater reduction in UI domain. There was no significant association between dose‐fractionation regimens or use of hydrogel spacer and the score changes in the three domains.

**TABLE 5:** Factors associated with early changes in each domain of the EPIC‐26 score over time

EPIC‐26 domain	Independent variable	Coefficient	*P*‐value^a^, ^b^
Bowel	Radiation modality: PBT	9.1	<.001
Urinary irritative/obstructive	Baseline UO score	−0.4	.01
	(Baseline UI score)*(Time)^c^	See footnote^c^	<.001
Urinary incontinence	Baseline BF score	−0.3	.05
	Baseline UO score	0.3	.003
	Baseline UI score	−0.4	<.001

^a^ The generalized estimating equations (GEE) were used to identify factors that were statistically significantly associated with changes in the scores over time. Independent covariance structure was selected for the GEE, based on the quasi‐information criterion (QIC).^8^ *P*‐values reflect a test of individual covariate effect. *P* < .05 is considered significant. Both QIC and *P*‐values were computed based on Wald test and robust standard errors.

^b^ Variables included in the GEE models were the type of radiation modality (PBT vs IMRT), dose‐fractionation regimen utilized (60 Gy/20 f vs 70.2 Gy/26 f vs 78 Gy/39 f), the use of hydrogel spacer to reduce radiation dose to the rectum (yes vs no), baseline BF, UO, and UI scores, age, PSA, Gleason score, T stage, and the use of ADT. We excluded race from this analysis because of the small number of non‐Caucasian patients. Indicator variables were included to distinguish radiation modality and the time of survey (the end of RT and 3 months post‐RT). Our model also included interaction terms between each variable and time indicator. Stepwise backward/forward variable selections were conducted for the model construction based on QIC. Statistically significant interactions were investigated in Simple Effect Tests to identify significance of main effects. Only those variables and interactions that were found statistically significant are tabulated.

^c^ Significant interaction was noted between baseline UI score and time. While a higher baseline UI score (ie less urinary incontinence) was associated with greater UO reduction at the end of RT (*P* = .016), there was no association between baseline UI score and UO score reduction at 3‐month post‐RT.

## 4 DISCUSSION

PBT has been increasingly used in clinical practice for treatment of clinically localized prostate carcinoma in recent years. Several dosimetric studies demonstrated that treatment with PBT resulted in less overall RT dose to the rectum and bladder than IMRT, while maintaining the intended dose to the prostate.^9^, ^10^, ^11^ These dosimetric advantages likely explain the less acute decrement in BF with PBT observed in our study.

Our findings on BF are different than two prior studies using Surveillance Epidemiology and End Results data to report claims‐based toxicities^12^, ^13^ which reported patients treated with PBT had higher rates of gastrointestinal toxicity compared to those treated with three‐dimensional conformal RT (3‐DCRT), IMRT, brachytherapy, and conservative management. However, these studies were based on Medicare claims and were subject to several confounding factors due to the use of Medicare claims as surrogates for relevant endpoints of treatment‐related toxicity and QOL instead of direct patient outcomes. Our BF findings are also different from those of the comparative study by Hoppe et al.^14^ Similar to ours, this study compared QOL outcomes, using EPIC‐26 questionnaire, between patients treated with IMRT and those receiving PBT. It reported no difference in the score changes in BF, UO, and UI domains at 6 months, 1 year, and 2 years post‐RT between the two cohorts. The discordant finding between this study and ours is likely, in part, due to the difference in a specific time point of QOL analysis. Hoppe et al examined the changes in QOL occurring at 6 months to 2 years post‐RT, whereas ours analyzed the change occurring at the end of RT and 3 months post‐RT. In addition, unlike ours, it had very diverse sources of QOL data, involving nine university‐affiliated hospitals for the IMRT QOL data and one institution for the PBT QOL data. This diverse source of QOL data, as well as the likelihood of heterogeneity in treatment planning and patient population, may have confounded its study findings.

Conversely, our study findings are consistent with another study which used prospectively collected QOL for the comparative analysis of QOL among patients treated with PBT, IMRT, or 3‐DCRT.^15^ In this study, patients treated with 3‐DCRT and IMRT had clinically meaningful decrements in bowel QOL at 2‐3 months from the start of RT, while those receiving PBT did not. Additionally, this study reported that the IMRT cohort had clinically meaningful decrements in UI and UO QOL at 2‐3 months from the start of RT, which were not observed in the PBT cohort.

There are various definitions of a clinically meaningful EPIC‐26 score change. In this study, we used the widely accepted definition of a clinical meaningful change as a score change >50% of the standard deviation of a baseline score.^5^, ^6^, ^7^ The minimally important differences (MID) can also be utilized as a threshold to define a clinically meaningful change. Using MID for clinical relevance, an EPIC‐26 score change of 4‐6 points, 5‐7 points, and 6‐9 points should be considered to be clinically meaningful in the BF, UO, and UI domains, respectively.^16^ By applying MID threshold to our cohort, the BF mean change from baseline to end of RT remains clinically meaningful for both the IMRT and PBT cohorts with greater clinically meaningful deterioration in the IMRT group (data not shown). This BF mean change remains clinically meaningful 3‐months post RT for the IMRT group; however, it becomes no longer clinically meaningful for the PBT group. The UO mean change from baseline to the end of RT is also clinically meaningful for both the IMRT and PBT groups, when using the MID threshold. The UO mean change from baseline to 3 months post RT, and the UI score change at both end of RT and 3 months post RT are not clinically meaningful, based on the MID threshold. Thus, the results of this study remain consistent, even when using an alternate definition of a clinically meaningful change.

There are several limitations in our study. First, our study is based on a non‐randomized comparison. Second, patients may have answered the EPIC‐26 questionnaire with preconceived bias based on the treatment modality they received. Third, the response rate of completing the EPIC‐26 questionnaire was not robust, dropping below 50% at 3 months post‐RT. Fourth, there were some differences in patient characteristics between our two cohorts with respect to dose‐fractionation regimens and the concomitant use of ADT. Based on the assessment of independent effects of multiple variables, dose‐fractionation regimens and ADT were not associated with the change in the scores of BF, UO, and UI domains over time in our study. Thus, it is unlikely that the difference in dose‐fractionation regimens and ADT use between the two cohorts would have impacted our study results. Fifth, there may be other unrecognized confounders affecting our study findings (eg the prevalence of irritable bowel symptoms at baseline and prostate volume). Sixth, the scope of our study is limited to the early phase of post‐RT. The comparative data on late toxicity and QOL change is clinically more meaningful in the day‐to‐day clinical practice, since the early toxicity and QOL change are often temporary and resolve over time. In addition, many radiation oncology centers do not have access to PBT, not all patients have PBT insurance coverage, and most centers charge more for PBT than IMRT. Late toxicity data will be essential in justifying the utilization of PBT for prostate cancer, given the challenges with access, insurance approval, and cost. Lastly, our study did not incorporate dosimetric data for the analysis of QOL change. It is well recognized that the volume of each normal organ (rectum and bladder) exposed to specific RT doses is a strong predictor of early and late toxicity.^17^, ^18^ Thus, the distribution of dose is likely an important factor in QOL outcomes and may be independent of RT modality.

## 5 CONCLUSION

There have been conflicting reports of QOL outcomes of patients treated with IMRT and PBT for localized prostate cancer. Our study serves to provide a single institution's comparative analysis between IMRT and PBT with respect to the changes in BF, UO, and UI domains of QOL in the early phase of post‐RT, using prospectively collected EPIC‐26. In our study, PBT had less early decrement in BF than IMRT, while there was no differential effect on UO and UI. Further studies, preferably a randomized trial, are needed to demonstrate whether there is any meaningful clinical difference in RT toxicity, QOL changes, and tumor control between these two modalities.

## CONFLICT OF INTEREST

Dr Davis reports personal fees and other from Boston Scientific, Inc during the conduct of the study; other from Pfizer, Inc, other from American Brachytherapy Society, and nonfinancial support from American Board of Radiology outside the submitted work.

## AUTHOR CONTRIBUTIONS

(I) Conception and design: All authors.

(II) Administrative support: None.

(III) Provision of study materials or patients: None.

(IV) Collection and assembly of data: None.

(V) Data analysis and interpretation: None.

(VI) Manuscript writing: All authors.

(VII) Final approval of manuscript: All authors.

## Data Availability

Research data are stored in an institutional repository and will be shared upon request to the corresponding author.

## APPENDIX 1

**TABLE A1:** Comparison of baseline characteristics between respondents and non‐respondents to the 3‐month EPIC‐26 questionnaire

Characteristics	Respondents (n=129)	Non‐respondents (n=133)	*P*‐value*
Age			
Mean (range), year	71.3 (44‐88)	70.8 (52‐85)	0.55
Age group, n (%)			0.64
<70	46 (35.7%)	55 (41.4%)	
70‐79	75 (58.1%)	71 (53.4%)	
≥80	8 (6.2%)	7 (5.3%)	
Race			
Race, n (%)			0.22
White	126 (97.7%)	122 (91.7%)	
Others	3 (2.3%)	8 (6.0%)	
Missing	0	3 (2.3%)	
Pre‐RT PSA			
Mean (range, ng/mL)	7.6 (0.1‐34.3)	7.5 (0.1‐41.7)	0.26
Group, n. (%)			0.67
<4	31 (24.0%)	39 (29.3%)	
4‐10	65 (50.4%)	63 (47.4%)	
>10	33 (25.6%)	31 (23.3%)	
Gleason score			
Group, n (%)			0.38
≤7	113 (87.6%)	111 (83.5%)	
>7	16 (12.4%)	22 (16.5%)	
T stage			
Group, n. (%)			0.17
T1	52 (40.3%)	65 (48.9%)	
T2	77 (59.7%)	68 (51.1%)	
Radiation modality			0.45
IMRT, n (%)	74 (57.4%)	83 (62.4%)	
PBT, n (%)	55 (42.6%)	50 (37.6%)	
Dose‐fractionation regimen			
n (%)			0.15
60 Gy/20 f	47 (36.4%)	61 (45.9%)	
70.2 Gy/26 f	68 (52.7%)	54 (40.6%)	
78Gy/39 f	14 (10.9%)	18 (13.5%)	
Hydrogel spacer			
Treated with hydrogel spacer, n (%)	56 (43.4%)	68 (51.1%)	0.22
Androgen deprivation therapy (ADT)			
Treated with ADT, n (%)	95 (73.6%)	103 (77.4%)	0.57
Baseline bowel score			
Mean (range)	93.4 (50.0‐100)	94.4 (29.2‐100)	0.38
Standard deviation	10.1	10.2	
Missing	2	4	
Baseline urinary irritative/obstructive score			
Mean (range)	84.4 (43.8‐100)	86.8 (31.3‐100)	0.23
Standard deviation	14.3	12.5	
Missing	3	8	
Baseline urinary incontinence score			
Mean (range)	87.5 (31.0‐99.5)	87.8 (22.8‐99.5)	0.46
Standard deviation	17.5	15.2	
Missing	1	1	

* *P*‐values were derived from Wilcoxon rank‐sum test for continuous variables and Fisher's exact test for categorical variables. *P*‐values reflect a test whether the means or the distributions were different between two cohorts. *P* < .05 is considered significant.
